# Pre-treatment IFN-γ levels are associated with chronic progression in patients with brucellosis: a retrospective study

**DOI:** 10.3389/fimmu.2026.1876492

**Published:** 2026-07-23

**Authors:** Jinhua Yuan, Qiuyan Chen, Qingcao Fei, Meiying Gu, Lina Ma, Xiangchun Ding

**Affiliations:** 1 Ningxia Medical University, Yinchuan, China; 2 Department of Infectious Disease, General Hospital of Ningxia Medical University, Yinchuan, China; 3 Infectious Disease Clinical Research Center of Ningxia, Yinchuan, China

**Keywords:** brucellosis, chronic progression, IFN-γ, restricted cubic spline, retrospective, risk prediction

## Abstract

**Background:**

Chronic brucellosis may lead to persistent symptoms and multisystem complications, but objective markers for identifying patients at high risk of chronic progression remain lacking. This study investigated the association between pre-treatment interferon-gamma (IFN-γ) levels and chronic progression in brucellosis.

**Methods:**

This single-center retrospective study included patients diagnosed with brucellosis at the General Hospital of Ningxia Medical University between January 2021 and June 2025. The primary outcome was progression to chronic brucellosis. Logistic regression was used to assess the association between pre-treatment IFN-γ levels and chronic progression risk. Restricted cubic spline (RCS) and subgroup analyses were performed to examine the dose-response relationship and the robustness of the findings. Candidate predictors were selected using complementary statistical and feature-selection approaches, and an exploratory baseline model was constructed to assess whether IFN-γ provided incremental predictive information.

**Results:**

Multivariable logistic regression showed that pre-treatment IFN-γ levels were independently and inversely associated with chronic progression risk. In the fully adjusted model, this association remained consistent when IFN-γ was analyzed as a continuous variable, a tertile-based categorical variable, or a binary variable using the cutoff derived from RCS analysis, supporting the robustness of the findings. RCS analysis demonstrated a significant nonlinear inverse association between IFN-γ levels and chronic progression risk, with an inflection point around 13.47. Subgroup analyses showed that this association was generally consistent across age, sex, exposure history, occupation, and comorbidity strata. Adding IFN-γ to the baseline model produced a modest but statistically significant increase in discrimination, with the AUC increasing from 0.855 (95% CI: 0.795–0.915) and 0.894 (95% CI: 0.847–0.942; ΔAUC = 0.039; P = 0.021). Improvements in net reclassification improvement (NRI) and integrated discrimination improvement (IDI) were also observed.

**Conclusions:**

Higher pre-treatment IFN-γ levels were independently associated with a lower risk of chronic progression in patients with acute brucellosis. IFN-γ may serve as a promising exploratory biomarker for early risk stratification of chronic progression in brucellosis. However, the prediction model was not validated, and its clinical utility requires confirmation in multicenter prospective cohorts.

## Introduction

Brucellosis is a globally prevalent zoonotic disease caused by Brucella species and continues to pose a substantial public health and socioeconomic burden, especially in low- and middle-income countries (1). After infection, Brucella may affect nearly all tissues and organs, leading to highly heterogeneous clinical manifestations ranging from asymptomatic infection to systemic disease with multisystem involvement (2). Acute brucellosis typically presents with fever, sweating, osteoarticular pain, and hepatosplenomegaly, and most patients respond well to timely and standardized therapy (3). Nevertheless, a proportion of patients develop persistent symptoms, recurrent episodes, and organ-specific complications despite standard treatment, ultimately progressing to chronic brucellosis (4).

Chronic brucellosis represents a major challenge in clinical practice. However, existing burden-of-disease studies on brucellosis differ substantially in their methodological approaches and underlying assumptions, and the reported proportion of chronic brucellosis varies across regions and study populations. Therefore, the global burden of chronic brucellosis remains difficult to estimate precisely (5). It has been reported that approximately 5%–15% of patients with acute brucellosis in China progress to the chronic stage (3). To date, no reliable laboratory-specific diagnostic markers are available, and diagnosis mainly depends on disease duration, clinical presentation, and clinical judgment (3). Standard treatment is based on combination antibiotic therapy, most commonly rifampin plus doxycycline, with cephalosporins or fluoroquinolones added in selected cases (6). Although prolonged treatment may reduce relapse or persistence, it does not completely prevent chronic progression (7). More importantly, objective tools for identifying patients at high risk of chronicity during the acute stage are still lacking.

As a facultative intracellular pathogen, Brucella is controlled primarily through Th1-mediated cellular immune responses (8). IFN-γ is a pivotal Th1 cytokine that promotes macrophage activation, enhances intracellular bacterial killing, and contributes to immune homeostasis during infection (8, 9). Impaired cell-mediated immunity has been reported in patients with chronic brucellosis (9), suggesting that IFN-γ may be associated with disease persistence and chronic progression.

However, previous studies on IFN-γ in brucellosis have mainly focused on gene expression and immunological mechanisms, while evidence on the association between pre-treatment IFN-γ levels and subsequent chronic progression remains limited, especially in clinical cohort settings (10, 11). Moreover, whether IFN-γ provides incremental predictive value beyond conventional clinical indicators has not been fully determined (12). Therefore, the primary objective of this study was to evaluate whether pre-treatment IFN-γ levels were clinically associated with subsequent chronic progression, rather than to infer a causal or etiologic relationship. The secondary objective was to explore whether IFN-γ could provide additional predictive value beyond conventional clinical and laboratory indicators.

## Methods

### Study design and participants

This was a single-center retrospective study. The study population consisted of hospitalized patients who were first diagnosed with brucellosis at the General Hospital of Ningxia Medical University between January 2021 and June 2025. All included patients presented with clinical manifestations consistent with brucellosis and were confirmed by serological testing or positive blood culture according to established diagnostic criteria cited in the literature (13). Eligible patients were adults (≥18 years), were in the acute stage of disease, received standardized treatment, completed at least one full treatment course, and underwent 6 months of follow-up. Follow-up information was obtained through outpatient visits and telephone follow-up. As shown in the study flowchart, only patients who completed the scheduled 6-month follow-up and had sufficient information to determine their outcome status were included in the final analytic. No outcome imputation was performed. No deaths were recorded in the final analytic. Patients who experienced complications during follow-up were retained if their 6-month outcome status was available and were classified according to the predefined diagnostic criteria for chronic brucellosis.

The exclusion criteria were as follows: (1) incomplete data on key variables, including IFN-γ and outcome variables; (2) an initial diagnosis of chronic brucellosis or latent infection; (3) concomitant tuberculosis, typhoid fever, paratyphoid fever, rheumatic fever, other natural focal diseases, or immunodeficiency; (4) ongoing long-term corticosteroid or immunosuppressive therapy; and (5) pregnancy or lactation.

**Figure 1:**
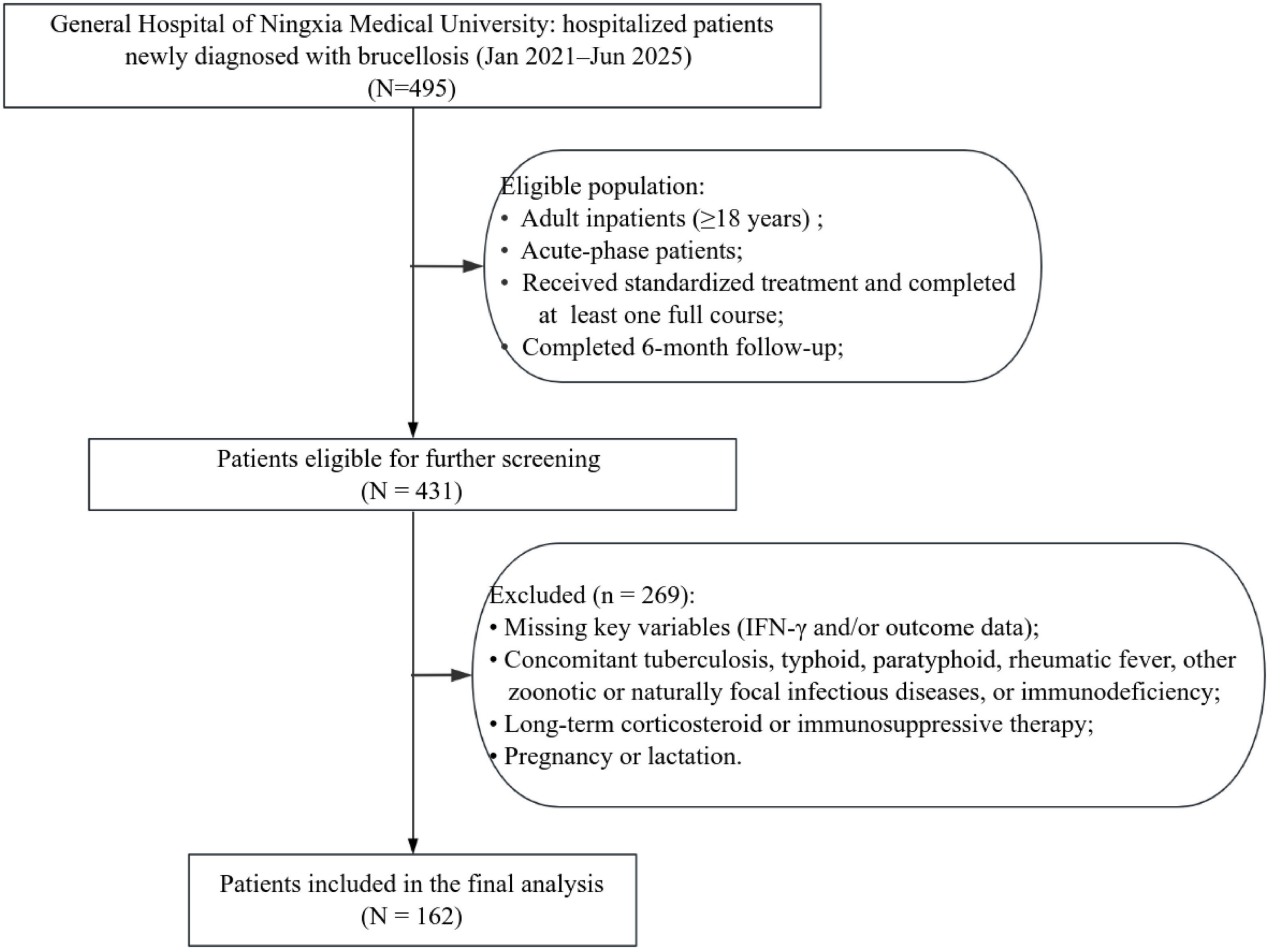
Flow diagram of participant selection.

The study flowchart is shown in **Figure 1**. A total of 495 newly diagnosed hospitalized patients were screened, 431 met the criteria for further evaluation, and 162 were ultimately included in the final analysis. The study protocol was approved by the Medical Ethics Committee of the General Hospital of Ningxia Medical University (approval No. KYLL-2025-0873), and all procedures were conducted in accordance with the Declaration of Helsinki.

### Outcome definition

The primary outcome of this study was progression to chronic brucellosis within 6 months. Chronic brucellosis was defined as a disease course lasting more than 6 months, with failure to achieve clinical cure and persistent fulfillment of the diagnostic criteria for brucellosis (3).

### Data collection and definitions

Baseline data were collected at the time of first confirmed diagnosis and before the initiation of any treatment. These data included demographic and epidemiological characteristics, including age, sex, exposure history, and occupation. Exposure history was defined as documented pre-onset exposure to potential sources of Brucella infection, including contact with livestock or animal products, occupational exposure related to animal husbandry, slaughtering, or veterinary work, contact with aborted animal materials, or consumption of unpasteurized dairy products. Comorbidities included hypertension, diabetes mellitus, and coronary heart disease. Clinical manifestations included fever, fatigue, sweating, osteoarticular pain, hepatomegaly, splenomegaly, and lymphadenopathy. Treatment regimens included RIF + DOX, DOX or RIF + FQs, DOX + RIF + FQs, and DOX + RIF + Ceph. Laboratory parameters included white blood cell count (WBC), platelet count (PLT), neutrophil count (Neu#), lymphocyte count (Lym#), serum albumin (ALB), C-reactive protein (CRP), erythrocyte sedimentation rate (ESR), serum ferritin (SF), and IFN-γ. All laboratory data were extracted from the hospital laboratory information system. Pre-treatment IFN-γ concentrations were measured in peripheral venous blood samples collected before initiation of antimicrobial therapy. The measurements were performed and reported by the hospital clinical laboratory according to standardized laboratory procedures. Results were expressed in pg/mL, and all assays were conducted under routine internal quality-control procedures.

Based on these laboratory parameters, several immune-, nutritional-, and inflammation-related indices were further calculated, including the systemic immune-inflammation index (SII), neutrophil-to-lymphocyte ratio (NLR), platelet-to-lymphocyte ratio (PLR), CRP-to-albumin ratio (CAR), prognostic nutritional index (PNI), and CALLY index. These indices were defined as follows: SII (14)=PLT (×10^9/L)×Neu# (×10^9/L)/Lym# (×10^9/L); NLR (14)=Neu# (×10^9/L)/Lym# (×10^9/L); PLR (14)=PLT (×10^9/L)/Lym# (×10^9/L); CAR (15)=CRP (mg/L)/ALB (g/L); PNI (16)=ALB (g/L) + 5×Lym# (×10^9/L); and CALLY (17)=ALB (g/L)×Lym# (×10^9/L)/CRP (mg/L).

### Statistical analysis

All statistical analyses were performed using R software (version 4.4.3). Baseline characteristics were summarized for the overall cohort and compared by outcome status, defined as no chronic progression versus progression to chronic brucellosis. As a supplementary descriptive analysis, patients were also stratified into tertiles according to pre-treatment IFN-γ levels. Continuous variables were presented as mean ± SD or median (IQR) and compared using two-sample t-test, Mann–Whitney U test, one-way ANOVA, or the Kruskal–Wallis test, as appropriate. Categorical variables were presented as n (%) and compared using the χ² test or Fisher’s exact test.

Logistic regression analysis was performed to evaluate the association between pre-treatment IFN-γ levels and the risk of chronic brucellosis using three models: a crude model, Model 1, and Model 2. Model 1 was adjusted for age, sex, exposure history, occupation, hypertension, diabetes mellitus, and coronary heart disease. Model 2 was further adjusted for fever, fatigue, sweating, osteoarticular pain, hepatomegaly, splenomegaly, lymphadenopathy, and treatment regimen in addition to the variables included in Model 1.

Restricted cubic spline (RCS) analysis was further performed to explore the dose-response relationship between IFN-γ levels and the risk of chronic progression. The cutoff value of 13.47 pg/mL was identified *post hoc* from the RCS curve as an approximate inflection point. It was not predefined and was therefore used only as an exploratory threshold in supplementary analyses. Subgroup analyses were performed according to age, sex, exposure history, occupation, and major comorbidities, and interaction tests were used to assess the consistency of the association across subgroups.

As an exploratory component of the predictive analysis, we developed a parsimonious baseline comparator model to evaluate the incremental predictive information associated with IFN-γ. Univariable logistic regression, least absolute shrinkage and selection operator (LASSO) regression, the Boruta algorithm, and recursive feature elimination (RFE) were used as complementary exploratory approaches for variable selection. Variance inflation factors (VIFs) were calculated to evaluate potential multicollinearity among candidate predictors. Based on the combined consideration of variable-selection results, multicollinearity, and clinical interpretability, a restricted set of predictors was retained for the baseline comparator model. IFN-γ was subsequently added to this model to examine its incremental discriminative value.

Model discrimination was evaluated using the area under the receiver operating characteristic curve (AUC), and differences in AUC between models were assessed using the DeLong test. Net reclassification improvement (NRI) and integrated discrimination improvement (IDI) were also calculated as exploratory measures of the incremental discriminative value of IFN-γ. It should be noted that cross-validation was used only for algorithm-specific parameter tuning and feature selection. Therefore, the reported AUC, NRI, and IDI values represent apparent performance estimates derived from the same study sample and may be subject to a degree of optimism.

## Results

### Baseline characteristics

Baseline characteristics according to chronic progression status are presented in **Table 1**. Among the 162 included patients, 35 (21.60%) progressed to chronic brucellosis. Demographic characteristics, exposure history, occupation, comorbidities, clinical manifestations, and treatment regimens were comparable between the two groups (all P > 0.05). Patients who progressed to chronic brucellosis had significantly lower pre-treatment IFN-γ levels than those who did not. Significant differences were also observed in ALB, CRP, ESR, SF, CAR, PNI, and CALLY (all P < 0.05).

**Table 1:** Baseline characteristics according to chronic progression status.

Characteristics	Overall (N=162)	Non-chronic brucellosis (N=127)	Chronic brucellosis (N=35)	*P*
Demographic characteristics				
Age(years), median (IQR)	49.50 (42.00, 58.75)	50.00 (42.50, 58.50)	49.00 (42.00, 59.00)	0.861
Sex, n (%)				0.832
Male	118 (72.84)	93 (73.23)	25 (71.43)	
Female	44 (27.16)	34 (26.77)	10 (28.57)	
History of exposure (Yes), n (%)	112 (69.14)	86 (67.72)	26 (74.29)	0.539
Occupation, n (%)				0.700
Farmer	66 (40.74)	53 (41.73)	13 (37.14)	
Non-farmer	96 (59.26)	74 (58.27)	22 (62.86)	
Comorbidity (Yes)				
Hypertension, n (%)	23 (14.20)	17 (13.39)	6 (17.14)	0.588
Diabetes, n (%)	11 (6.79)	9 (7.09)	2 (5.71)	>0.999
Coronary disease, n (%)	8 (4.94)	5 (3.94)	3 (8.57)	0.372
Clinical manifestations				
Fever, n (%)	126 (77.78)	95 (74.80)	31 (88.57)	0.108
Fatigue, n (%)	136 (83.95)	105 (82.68)	31 (88.57)	0.603
Hyperhidrosis, n (%)	87 (53.70)	72 (56.69)	15 (42.86)	0.181
Osteoarthritis, n (%)	106 (65.43)	81 (63.78)	25 (71.43)	0.430
Hepatomegaly, n (%)	7 (4.32)	7 (5.51)	0 (0.00)	0.348
Splenomegaly, n (%)	29 (17.90)	25 (19.69)	4 (11.43)	0.326
Lymphadenopathy, n (%)	15 (9.26)	13 (10.24)	2 (5.71)	0.528
Immunonutritional-inflammatory indices				
WBC(×10^9/L), median (IQR)	5.29 (3.89, 7.54)	5.16 (3.68, 7.33)	6.18 (4.58, 7.62)	0.090
PLT(×10^9/L), mean ± SD	217.79 ± 98.79	215.61 ± 100.99	225.70 ± 91.30	0.574
Neu#(×10^9/L), median (IQR)	2.94 (2.01, 4.70)	2.77 (1.95, 4.52)	3.69 (2.65, 4.80)	0.170
Lym#(×10^9/L), median (IQR)	1.64 (1.20, 2.15)	1.62 (1.14, 2.10)	1.82 (1.42, 2.36)	0.108
ALB(g/L), median (IQR)	36.06 (32.39, 39.85)	35.40 (31.90, 37.95)	39.70 (35.67, 42.66)	<0.001
CRP(mg/L), median (IQR)	31.20 (13.48, 71.58)	34.10 (18.70, 73.00)	12.00 (6.00, 34.95)	<0.001
ESR(mm/h), median (IQR)	15.00 (7.00, 31.75)	18.00 (8.00, 33.00)	12.00 (3.00, 22.00)	0.030
SF(ng/mL), median (IQR)	530.50 (377.00, 927.00)	723.00 (418.00, 1010.00)	313.00 (148.00, 449.50)	<0.001
IFN-γ, median (IQR)	13.47 (4.62, 32.77)	20.05 (5.78, 38.42)	6.20 (2.22, 12.20)	<0.001
SII, median (IQR)	387.98 (208.26, 667.18)	362.92 (207.57, 601.33)	452.35 (225.96, 730.40)	0.339
NLR, median (IQR)	1.99 (1.21, 2.76)	1.97 (1.24, 2.76)	2.05 (1.16, 2.68)	0.971
PLR, median (IQR)	122.85 (87.01, 186.92)	123.12 (82.50, 183.67)	118.49 (91.07, 195.98)	0.872
CAR, median (IQR)	0.89 (0.34, 1.86)	0.96 (0.52, 2.14)	0.29 (0.15, 0.91)	<0.001
PNI, median (IQR)	43.95 (39.94, 49.48)	43.31 (39.55, 47.82)	48.35 (43.23, 52.72)	0.002
CALLY, median (IQR)	1.84 (0.86, 4.60)	1.53 (0.73, 3.15)	6.33 (1.92, 13.39)	<0.001
**Treatment regimen, n (%)**				0.898
RIF + DOX	61 (37.65)	47 (37.01)	14 (40.00)	
DOX or RIF + FQs	11 (6.79)	8 (6.30)	3 (8.57)	
DOX + RIF + FQs	77 (47.53)	61 (48.03)	16 (45.71)	
DOX + RIF + Ceph	13 (8.02)	11 (8.66)	2 (5.71)	

Patients were divided into a Non-chronic brucellosis group and a chronic brucellosis group according to their outcome status. The chronic brucellosis group included patients who progressed to chronic brucellosis during follow-up. Continuous variables with normal distribution are presented as mean ± SD and compared using two-sample t-test; non-normally distributed continuous variables are presented as median (IQR) and compared using the Wilcoxon rank-sum test. Categorical variables are presented as n (%) and compared using Fisher’s exact test. WBC, white blood cell count; PLT, platelet count; Neu#, neutrophil count; Lym#, lymphocyte count; ALB, albumin; CRP, C-reactive protein; ESR, erythrocyte sedimentation rate; SF, serum ferritin; IFN-γ, interferon-gamma; SII, systemic immune-inflammation index; NLR, neutrophil-to-lymphocyte ratio; PLR, platelet-to-lymphocyte ratio; CAR, C-reactive protein-to-albumin ratio; PNI, prognostic nutritional index; CALLY, C-reactive protein–albumin–lymphocyte index; RIF, rifampicin; DOX, doxycycline; FQs, fluoroquinolones; Ceph, cephalosporins.

As a supplementary descriptive analysis, baseline characteristics by tertiles of pre-treatment IFN-γ levels are shown in **Supplementary Table 1**. Most clinical and epidemiological variables were comparable across tertiles, while WBC, Neu#, CRP, ESR, SF, CAR, and CALLY differed significantly (all P < 0.05).

### Association between IFN-γ and chronic progression of brucellosis

When stratified by tertiles of pre-treatment IFN-γ levels, the incidence of chronic progression was 36.36% (20/55), 26.42% (14/53), and 1.85% (1/54) in the T1, T2, and T3 groups, respectively, showing a statistically significant difference (P < 0.001). These findings suggest that higher IFN-γ levels were associated with a lower risk of chronic progression (**Table 2**). However, because only one patient in the highest IFN-γ tertile progressed to chronic brucellosis, the estimate for this group may be unstable and should be interpreted with caution.

**Table 2:** Incidence of chronic brucellosis according to tertiles of pre-treatment IFN-γ levels.

Characteristics	Overall (N = 162)	T1 (N = 55)	T2 (N = 53)	T3 (N = 54)	P
Chronic brucellosis, n (%)					<0.001
No	127 (78.40)	35 (63.64)	39 (73.58)	53 (98.15)	
Yes	35 (21.60)	20 (36.36)	14 (26.42)	1 (1.85)	

Patients were categorized into tertiles according to pre-treatment IFN-γ levels: T1 ≤ 8.44 pg/mL, T2 > 8.44 to ≤ 28.22 pg/mL, and T3 > 28.22 pg/mL. Chronic brucellosis was defined as a disease course lasting more than 6 months, with failure to achieve clinical cure and persistent fulfillment of the diagnostic criteria for brucellosis. Data are presented as n (%). The P value was calculated using the χ² test or Fisher’s exact test, as appropriate. IFN-γ, interferon-gamma.

**Figure 2:**
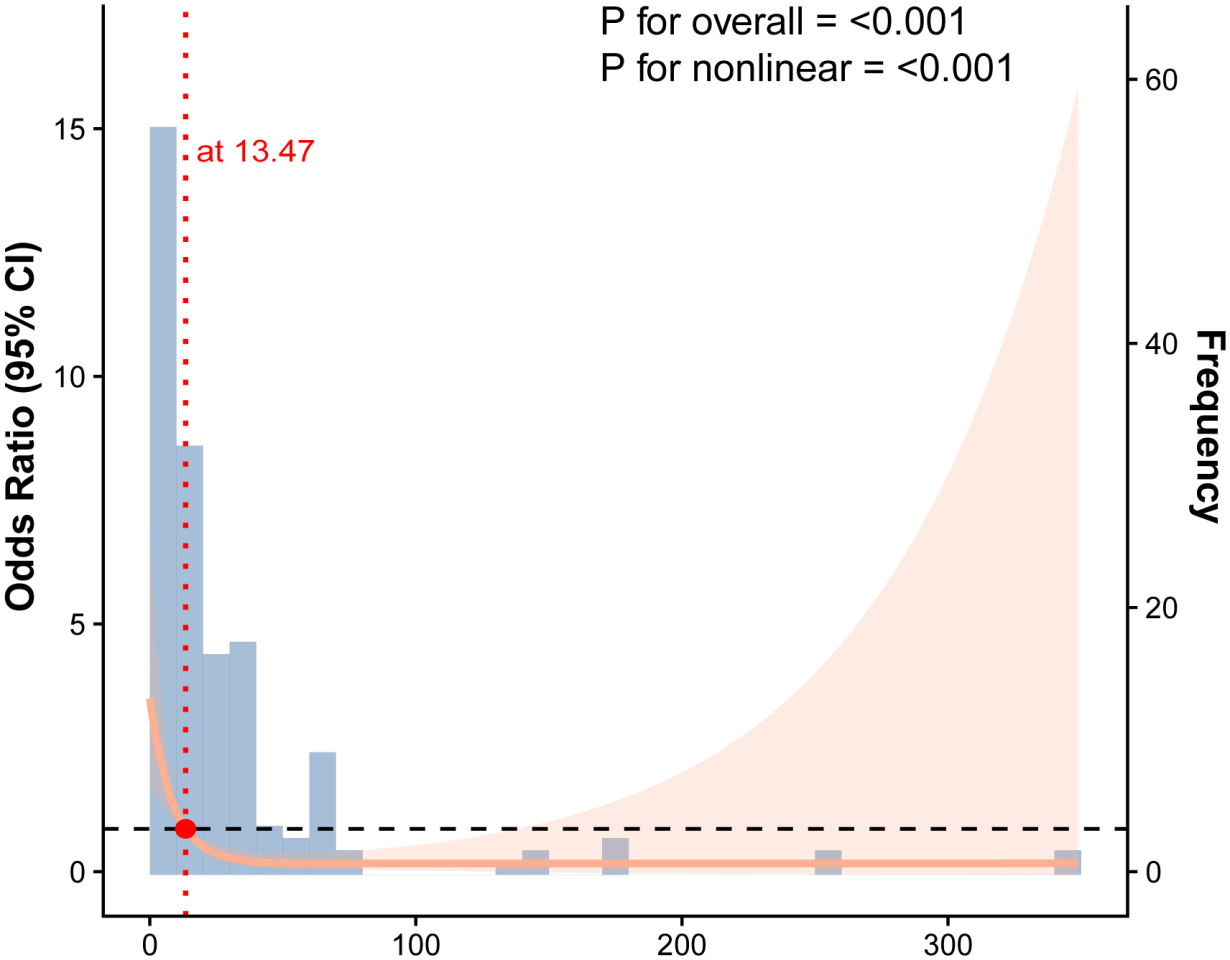
RCS analysis of the association between IFN-γ levels and progression to chronic brucellosis. The solid line indicates adjusted odds ratios and the shaded area indicates 95% confidence intervals. The model was adjusted for demographic characteristics, comorbidities, clinical manifestations, and treatment regimen. An inflection point was observed at approximately 13.47.

Restricted cubic spline (RCS) analysis demonstrated a significant nonlinear dose-response relationship between pre-treatment IFN-γ levels and the risk of chronic brucellosis (P for overall < 0.001; P for nonlinear < 0.001). As shown in **Figure 2**, the risk of chronic progression generally decreased with increasing IFN-γ levels, with an apparent inflection point around 13.47, beyond which the decline in risk became more gradual. As this threshold was data-derived from the current data, it should be interpreted as an exploratory cutoff.

The results of logistic regression analyses are presented in **Table 3**. When IFN-γ was analyzed as a continuous variable, it was significantly and inversely associated with the risk of chronic brucellosis, and this association remained statistically significant after full adjustment (Model 2: OR = 0.916, 95% CI: 0.872–0.961, P < 0.001). Using the T1 group as the reference, the T3 group showed a significantly lower risk of chronic progression (Model 2: OR = 0.026, 95% CI: 0.003–0.215, P < 0.001). Furthermore, when IFN-γ was dichotomized using 13.47 as the cutoff, the higher IFN-γ group (G2) had a markedly lower risk of chronic progression than the lower IFN-γ group (G1) (Model 2: OR = 0.048, 95% CI: 0.013–0.187, P < 0.001). Trend tests were statistically significant across all models (all P for trend < 0.001).

**Table 3:** Association of pre-treatment IFN-γ with chronic progression in logistic regression models.

Variable	Crude		Model1		Model2	
	OR(95%CI)	P	OR(95%CI)	P	OR(95%CI)	P
IFN-γ	0.929 (0.893, 0.966)	<0.001	0.925 (0.887, 0.964)	<0.001	0.916 (0.872, 0.961)	<0.001
T1 (ref)						
T2	0.628 (0.276, 1.428)	0.267	0.574 (0.243, 1.358)	0.207	0.492 (0.181, 1.338)	0.165
T3	0.033 (0.004, 0.257)	0.001	0.031 (0.004, 0.245)	0.001	0.026 (0.003, 0.215)	<0.001
G1 (ref)						
G2	0.059 (0.017, 0.203)	<0.001	0.055 (0.016, 0.194)	<0.001	0.048 (0.013, 0.187)	<0.001
P trend		<0.001		<0.001		<0.001

IFN-γ was analyzed as a continuous variable, a tertile-based categorical variable, and a binary variable using the cutoff identified by RCS. For tertile-based analyses, T1 (≤ 8.44) was used as the reference group, with T2 defined as > 8.44 to ≤ 28.22 and T3 as > 28.22. For binary analyses, G1 (≤ 13.47) was used as the reference group and G2 was defined as > 13.47. The crude model was unadjusted. Model 1 was adjusted for age, sex, history of exposure, occupation, hypertension, diabetes, and coronary disease. Model 2 was further adjusted for fever, fatigue, hyperhidrosis, osteoarthritis, hepatomegaly, splenomegaly, lymphadenopathy, and treatment regimen. P for trend was calculated across IFN-γ tertiles. IFN-γ, interferon-gamma.

### Subgroup analyses

**Figure 3:**
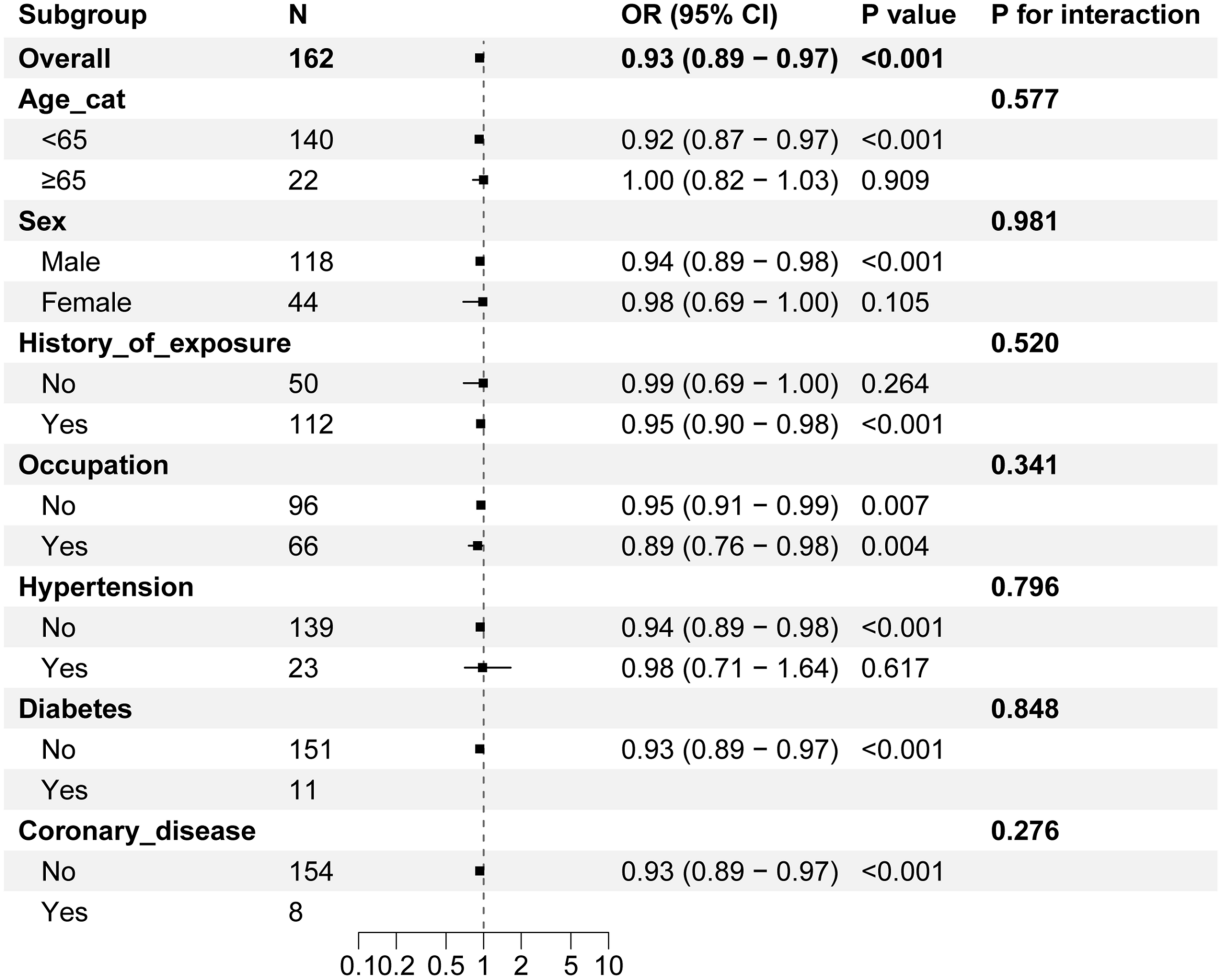
Subgroup analyses of the association between pre-treatment IFN-γ levels and chronic progression risk. ORs and 95% CIs were estimated from adjusted logistic regression models with IFN-γ analyzed as a continuous variable. Models were adjusted for the same covariates as the fully adjusted model, except for the stratification variable. P values for interaction were calculated to assess whether the association differed across subgroup strata.

Subgroup analyses were performed according to baseline characteristics, including age, sex, exposure history, occupation, and comorbidities (hypertension, diabetes, and coronary heart disease). The inverse association between IFN-γ levels and the risk of chronic progression remained generally consistent across all subgroups. No significant interactions were observed (all P for interaction > 0.05), indicating that the association was stable and was not significantly modified by these factors (**Figure 3**).

### Exploratory construction of the baseline prediction model

The results of logistic regression analyses for candidate variables are shown in **Table 4**. After adjustment for age, sex, exposure history, occupation, hypertension, diabetes, and coronary heart disease, ALB, CRP, SF, CAR, PNI, and CALLY remained significantly associated with the study outcome (all P < 0.05), suggesting that these indicators were important candidate variables for subsequent baseline model development.

**Table 4:** Logistic regression analysis of candidate predictors for chronic progression.

Variable	Crude		Adjusted	
	OR (95%CI)	P	OR (95%CI)	P
Fever	2.611 (0.855, 7.966)	0.092	2.858 (0.888, 9.196)	0.078
Fatigue	1.624 (0.520, 5.068)	0.404	1.981 (0.602, 6.525)	0.261
Hyperhidrosis	0.573 (0.269, 1.220)	0.149	0.589 (0.269, 1.292)	0.187
Osteoarthritis	1.420 (0.627, 3.216)	0.401	1.377 (0.584, 3.246)	0.464
Hepatomegaly	0.000 (0.000, Inf)	0.987	0.000 (0.000, Inf)	0.986
Splenomegaly	0.526 (0.170, 1.629)	0.266	0.482 (0.146, 1.585)	0.229
Lymphadenopathy	0.531 (0.114, 2.475)	0.421	0.477 (0.097, 2.350)	0.363
WBC	1.065 (0.939, 1.208)	0.330	1.062 (0.926, 1.218)	0.392
PLT	1.001 (0.997, 1.005)	0.592	1.001 (0.997, 1.005)	0.689
Neu#	1.040 (0.899, 1.202)	0.600	1.031 (0.884, 1.204)	0.695
Lym#	1.328 (0.833, 2.118)	0.233	1.330 (0.814, 2.174)	0.255
ALB	1.144 (1.058, 1.236)	<0.001	1.161 (1.068, 1.261)	<0.001
CRP	0.974 (0.957, 0.990)	0.002	0.972 (0.954, 0.989)	0.001
ESR	0.978 (0.953, 1.002)	0.077	0.977 (0.952, 1.002)	0.076
SF	0.995 (0.994, 0.997)	<0.001	0.995 (0.993, 0.997)	<0.001
SII	1.000 (1.000, 1.000)	0.771	1.000 (0.999, 1.000)	0.753
NLR	0.973 (0.847, 1.118)	0.701	0.965 (0.831, 1.121)	0.643
PLR	0.999 (0.994, 1.003)	0.602	0.999 (0.994, 1.003)	0.562
CAR	0.344 (0.184, 0.643)	<0.001	0.321 (0.167, 0.616)	<0.001
PNI	1.094 (1.034, 1.157)	0.002	1.105 (1.041, 1.174)	0.001
CALLY	1.179 (1.086, 1.280)	<0.001	1.196 (1.095, 1.306)	<0.001

The crude model was unadjusted, whereas the adjusted model was adjusted for age, sex, history of exposure, occupation, hypertension, diabetes, and coronary disease. WBC, white blood cell count; PLT, platelet count; Neu#, neutrophil count; Lym#, lymphocyte count; ALB, albumin; CRP, C-reactive protein; ESR, erythrocyte sedimentation rate; SF, serum ferritin; SII, systemic immune-inflammation index; NLR, neutrophil-to-lymphocyte ratio; PLR, platelet-to-lymphocyte ratio; CAR, C-reactive protein-to-albumin ratio; PNI, prognostic nutritional index; CALLY, C-reactive protein–albumin–lymphocyte index.

**Figure 4:**
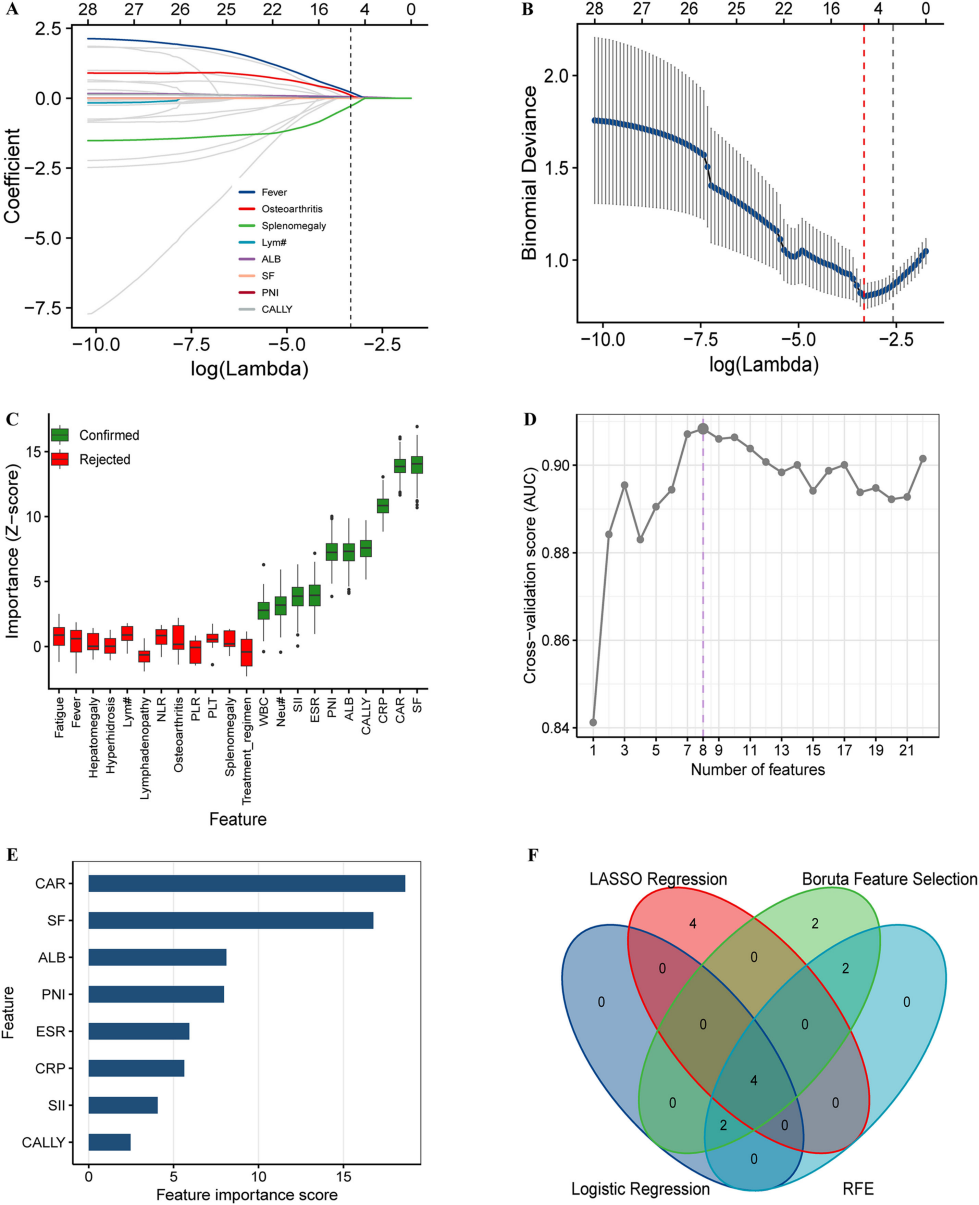
Feature selection process for candidate predictors used to construct the baseline prediction model. **(A)** LASSO coefficient profiles showing shrinkage of regression coefficients as the penalty parameter log(λ) changes. **(B)** Ten-fold cross-validation plot for LASSO regression used to determine the optimal penalty parameter; eight candidate features were retained at λ.min = 0.0359. **(C)** Boruta algorithm results, with green indicating confirmed important features and red indicating rejected features. **(D)** Cross-validated AUC values for different numbers of features in recursive feature elimination (RFE), showing the optimal model performance when eight features were included. **(E)** The eight important features selected by RFE and their importance ranking. **(F)** Venn diagram summarizing features selected by LASSO, Boruta, RFE, and logistic regression.

Multiple feature selection methods were then applied to optimize variable selection for the baseline model, and the results are shown in **Figure 4**. LASSO regression identified 8 candidate features after 10-fold cross-validation for optimal penalty selection, including fever, osteoarticular pain, splenomegaly, Lym#, ALB, SF, PNI, and CALLY (**Figures 4A, B**). The Boruta algorithm identified 10 important variables, namely SF, CAR, CRP, CALLY, PNI, ALB, ESR, SII, Neu#, and WBC (**Figure 4C**). RFE analysis showed that the model achieved optimal cross-validated AUC when 8 features were included, namely CAR, SF, ALB, PNI, ESR, CRP, SII, and CALLY (**Figures 4D, E**).

By integrating logistic regression results with three machine learning-based feature selection methods, ALB, SF, PNI, and CALLY were initially identified as candidate predictors (**Figure 4F**). Pairwise correlation and VIF analyses showed substantial collinearity between ALB and PNI (**Supplementary Tables 2**, **3**). Because PNI incorporates albumin-related information, ALB was excluded from the final model. After excluding ALB, all remaining variables had VIF values below 5 (**Supplementary Table 4**). Thus, SF, PNI, and CALLY were retained as core predictors for the exploratory baseline prediction model.

### Incremental predictive value of IFN-γ for chronic brucellosis

**Figure 5:**
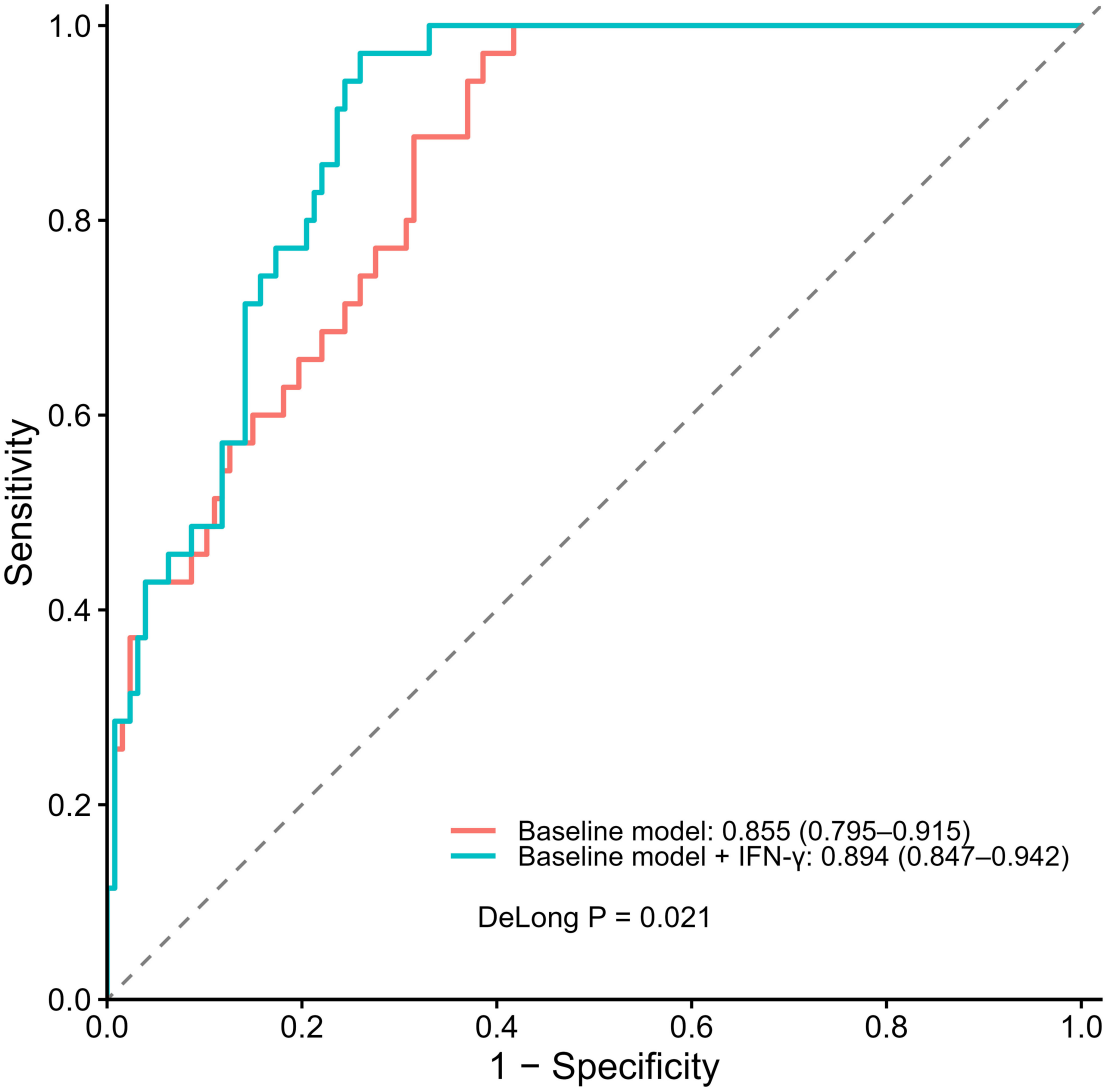
Comparison of ROC curves for the baseline model and the baseline model plus IFN-γ. The addition of IFN-γ improved the model’s discriminative performance.

A baseline prediction model was constructed based on the selected core predictors, yielding an area under the receiver operating characteristic curve (AUC) of 0.855 (0.795–0.915). After further incorporation of IFN-γ, the AUC increased to 0.894 (0.847-0.942), and the overall ROC performance was superior to that of the baseline model alone. The DeLong test showed that the difference in AUC between the two models was statistically significant (P = 0.020), indicating that the addition of IFN-γ moderately improved the model’s discriminative ability for chronic progression of brucellosis (**Figure 5**; **Table 5**).

**Table 5:** Discriminatory power of various models and incremental value of IFN-γ.

Model	AUC(95% CI)	P1	NRI(95% CI)	P2	IDI(95% CI)	P3
Baseline model	0.855 (0.795-0.915)					
Baseline model + IFN-γ	0.894 (0.847-0.942)	0.021	0.764 (0.353-1.067)	<0.001	0.071 (0.023-0.143)	0.020

NRI, net reclassification improvement; IDI, integrated discrimination improvement; P1, P value from DeLong test comparing AUCs; P2, P value for NRI; P3, P value for IDI. The baseline model was constructed using core predictors (SF, PNI, and CALLY), and the incremental model included both the baseline model and pre-treatment IFN-γ.

Further incremental performance analyses showed that, after adding IFN-γ to the baseline model, the NRI was 0.764 (95% CI: 0.353–1.067; P < 0.001), and the IDI was 0.071 (95% CI: 0.023–0.143; P = 0.020). These findings indicate that IFN-γ not only improved the overall discriminatory performance of the model but also enhanced risk reclassification, thereby providing additional predictive value for chronic progression in brucellosis (**Table 5**).

## Discussion

In this study, we found that pre-treatment IFN-γ levels were independently and inversely associated with the subsequent risk of progression to chronic brucellosis in patients with acute brucellosis. This association remained robust regardless of whether IFN-γ was analyzed as a continuous variable, a tertile-based categorical variable, or a binary variable defined according to the cutoff identified by RCS analysis. Moreover, RCS analysis revealed a significant nonlinear dose-response relationship between IFN-γ and the risk of chronic progression, rather than a simple linear association. Subgroup analyses further showed no significant interactions with age, sex, exposure history, occupation, or comorbidities, suggesting that this relationship was generally consistent across different populations. More importantly, the addition of IFN-γ to the conventional clinical model improved discrimination for chronic brucellosis, indicating that IFN-γ was not only associated with disease chronicity but also had may provide additional prognostic information for exploratory risk stratification.

Our findings are biologically plausible. The interaction between Brucella and the host immune system is critical in determining whether infection is cleared or progresses to chronic disease (18). Protective immunity against Brucella relies predominantly on cell-mediated immune responses, particularly those involving activated macrophages, dendritic cells, and T lymphocytes (19). As a key effector cytokine in the Th1 immune response, IFN-γ plays a central role in early immune control of Brucella infection by activating macrophages, enhancing phagocytic and bactericidal activity, promoting antigen presentation, and sustaining cellular immune effector functions (20, 21). In fact, our findings are highly consistent with previous reports (12). Higher pre-treatment IFN-γ levels may indicate that the host has already mounted a more effective anti-infective immune response during the early stage of disease, thereby helping to limit persistent intracellular survival of the pathogen and reducing the risk of prolonged disease and chronic progression (22, 23). In contrast, lower IFN-γ levels may reflect insufficient cell-mediated immunity, resulting in inadequate clearance of Brucella and an increased likelihood of chronicity (22, 23). Therefore, the inverse association observed in this study is not only statistically significant but also consistent with the immunopathophysiological features of brucellosis.

Notably, this study went beyond simply identifying an association and further evaluated the clinical predictive value of IFN-γ. The baseline model constructed using core predictors selected through multiple machine learning-based feature selection methods already showed good discriminative ability (24). After incorporation of IFN-γ, the model AUC further improved, and the DeLong test, NRI, and IDI all demonstrated statistically significant incremental value. These findings suggest that the information provided by IFN-γ cannot be fully captured by conventional clinical characteristics or routine inflammatory and nutritional indicators. Instead, IFN-γ appears to offer additional insight into chronic progression risk from the perspective of host cellular immune response. From a clinical standpoint, identifying patients with low IFN-γ levels at the initial stage may help optimize follow-up strategies, strengthen treatment-response assessment, and support individualized management.

In addition, we observed significant differences among IFN-γ groups in several inflammation-related indicators, including WBC, Neu#, CRP, ESR, SF, CAR, and CALLY. Overall, IFN-γ may not merely reflect the intensity of systemic inflammation, but rather the host immune response status against Brucella infection (25). Importantly, even after adjustment for demographic characteristics, clinical manifestations, comorbidities, and treatment regimens, IFN-γ remained independently associated with the risk of chronic progression. This suggests that its role is not entirely explained by general inflammatory status, but may more specifically reflect protective anti-Brucella cellular immunity. This may also explain why IFN-γ continued to provide additional predictive information beyond multiple established inflammatory and nutritional markers.

This study has several strengths. First, it focused on pre-treatment IFN-γ levels measured during the acute stage, which shifts the window for risk identification to an earlier phase of disease and better aligns with the clinical need for early warning and intervention. Second, logistic regression, RCS analysis, and subgroup analyses evaluated the association between IFN-γ and chronic progression from complementary perspectives. The additional feature-selection analyses were exploratory and were used only to examine the potential incremental information provided by IFN-γ; they were not intended to establish a validated prediction model. Finally, by using chronic progression as a clinically meaningful outcome, this study emphasized not only statistical association but also the potential value of IFN-γ in risk stratification, thereby increasing the clinical relevance of the results.

## Limitations

Several limitations should be acknowledged. First, this was a single-center retrospective study primarily involving hospitalized patients, with a relatively limited sample size, which may have introduced selection bias and restricted the generalizability of the findings. Therefore, the conclusions should be interpreted with caution when extrapolated to outpatient, mild, or community-based populations with brucellosis. Second, IFN-γ was measured only before the initiation of treatment, and the interval between symptom onset and blood sampling may have varied across patients. As a result, we were unable to assess dynamic changes in IFN-γ during treatment or to determine their temporal relationship with subsequent clinical outcomes. Third, the limited number of chronic progression events may have reduced the stability of the predictive analysis and increased the risk of overfitting, particularly because multiple data-driven feature-selection methods were used. Since variable selection and model fitting were performed in the same cohort without formal internal or external validation, the reported AUC, NRI, and IDI represent apparent in-sample estimates that may be optimistic. Therefore, the predictive analysis should be regarded as exploratory and hypothesis-generating rather than as evidence of a validated prediction model. Fourth, although exploratory prediction analyses were performed, the final prediction model was not formally subjected to internal or external validation. Therefore, its stability and generalizability remain to be confirmed in larger, multicenter, prospective cohort studies.

## Conclusions

In conclusion, higher pre-treatment IFN-γ levels were independently associated with a lower risk of chronic progression in patients with acute brucellosis. Exploratory in-sample analyses suggested that IFN-γ may provide additional prognostic information beyond selected clinical and laboratory indicators. However, owing to the limited number of outcome events, data-driven variable selection, and the absence of formal internal or external validation, the reported predictive performance may be optimistic. Larger multicenter prospective studies with appropriate validation are required before IFN-γ can be used for clinical risk stratification.

## Data Availability

The raw data supporting the conclusions of this article will be made available by the authors, without undue reservation.
