# Supplementary material for: Pre-treatment IFN-γ levels are associated with chronic progression in patients with brucellosis: a retrospective study
Source: Front Immunol. 2026 Jul 23;17:1876492. doi: 10.3389/fimmu.2026.1876492 (PMC13441725; doi:10.3389/fimmu.2026.1876492)
Supplement: Supplementary file 1 [file Table1.docx]

| Supplementary Table 1. Baseline characteristics according to tertiles of pre-treatment IFN-γ levels | | | | | | | |  |
| --- | --- | --- | --- | --- | --- | --- | --- | --- |
| **Characteristics** | **T1** (N=55) | | **T2** (N=53) | | **T3**  (N=54) | | **P** |  |
| **Demographic characteristics** | | | | | | | |  |
| Age(years), median (IQR) | 51.00 (42.00, 60.00) | | 49.00 (41.00, 55.00) | | 51.00 (43.00, 60.00) | | 0.643 |  |
| Sex, n (%) |  | |  | |  | | 0.326 |  |
| Male | 44 (80.00) | | 36 (67.92) | | 38 (70.37) | |  |  |
| Female | 11 (20.00) | | 17 (32.08) | | 16 (29.63) | |  |  |
| History of exposure, n (%) |  | |  | |  | | 0.459 |  |
| No | 18 (32.73) | | 13 (24.53) | | 19 (35.19) | |  |  |
| Yes | 37 (67.27) | | 40 (75.47) | | 35 (64.81) | |  |  |
| Occupation, n (%) |  | |  | |  | | 0.859 |  |
| Non-farmer | 34 (61.82) | | 30 (56.60) | | 32 (59.26) | |  |  |
| Farmer | 21 (38.18) | | 23 (43.40) | | 22 (40.74) | |  |  |
| **Comorbidity** |  | |  | |  | |  |  |
| Hypertension, n (%) |  | |  | |  | | 0.564 |  |
| No | 45 (81.82) | | 47 (88.68) | | 47 (87.04) | |  |  |
| Yes | 10 (18.18) | | 6 (11.32) | | 7 (12.96) | |  |  |
| Diabetes, n (%) |  | |  | |  | | 0.387 |  |
| No | 49 (89.09) | | 51 (96.23) | | 51 (94.44) | |  |  |
| Yes | 6 (10.91) | | 2 (3.77) | | 3 (5.56) | |  |  |
| Coronary disease, n (%) |  | |  | |  | | 0.076 |  |
| No | 50 (90.91) | | 50 (94.34) | | 54 (100.00) | |  |  |
| Yes | 5 (9.09) | | 3 (5.66) | | 0 (0.00) | |  |  |
| **Clinical manifestations** |  |  | |  | |  | |  |
| Fever, n (%) |  | |  | |  | | 0.520 |  |
| No | 15 (27.27) | | 11 (20.75) | | 10 (18.52) | |  |  |
| Yes | 40 (72.73) | | 42 (79.25) | | 44 (81.48) | |  |  |
| Fatigue, n (%) |  | |  | |  | | 0.127 |  |
| No | 13 (23.64) | | 5 (9.43) | | 8 (14.81) | |  |  |
| Yes | 42 (76.36) | | 48 (90.57) | | 46 (85.19) | |  |  |
| Hyperhidrosis, n (%) |  | |  | |  | | 0.054 |  |
| No | 32 (58.18) | | 24 (45.28) | | 19 (35.19) | |  |  |
| Yes | 23 (41.82) | | 29 (54.72) | | 35 (64.81) | |  |  |
| Osteoarthritis, n (%) |  | |  | |  | | 0.794 |  |
| No | 19 (34.55) | | 20 (37.74) | | 17 (31.48) | |  |  |
| Yes | 36 (65.45) | | 33 (62.26) | | 37 (68.52) | |  |  |
| Hepatomegaly, n (%) |  | |  | |  | | 0.270 |  |
| No | 54 (98.18) | | 49 (92.45) | | 52 (96.30) | |  |  |
| Yes | 1 (1.82) | | 4 (7.55) | | 2 (3.70) | |  |  |
| Splenomegaly, n (%) |  | |  | |  | | 0.471 |  |
| No | 43 (78.18) | | 43 (81.13) | | 47 (87.04) | |  |  |
| Yes | 12 (21.82) | | 10 (18.87) | | 7 (12.96) | |  |  |
| Lymphadenopathy, n (%) |  | |  | |  | | 0.751 |  |
| No | 50 (90.91) | | 47 (88.68) | | 50 (92.59) | |  |  |
| Yes | 5 (9.09) | | 6 (11.32) | | 4 (7.41) | |  |  |
| **Immunonutritional-inflammatory indices** | | | | | | | |  |
| WBC(×10^9/L), median (IQR) | 6.18 (4.55, 7.82) | | 4.61 (3.75, 7.72) | | 4.95 (3.64, 6.71) | | 0.027 |  |
| PLT(×10^9/L), mean ± SD | 226.10 ± 96.75 | | 215.58 ± 82.83 | | 211.49 ± 115.17 | | 0.730 |  |
| Neu#(×10^9/L), median (IQR) | 3.60 (2.21, 5.10) | | 2.69 (1.84, 4.38) | | 2.70 (2.02, 4.09) | | 0.048 |  |
| Lym#(×10^9/L), median (IQR) | 1.72 (1.19, 2.23) | | 1.75 (1.22, 2.31) | | 1.60 (1.20, 1.86) | | 0.359 |  |
| ALB(g/L), median (IQR) | 36.56 (34.00, 41.20) | | 36.20 (32.39, 40.40) | | 35.39 (31.90, 37.80) | | 0.165 |  |
| CRP(mg/L), median (IQR) | 33.80 (18.80, 73.60) | | 19.70 (8.02, 40.00) | | 34.95 (16.00, 72.80) | | 0.010 |  |
| ESR(mm/h), median (IQR) | 22.00 (11.00, 41.00) | | 16.00 (7.00, 31.00) | | 8.50 (6.00, 21.00) | | 0.002 |  |
| SF(ng/mL), median (IQR) | 413.00 (242.00, 529.00) | | 622.00 (377.00, 1,010.00) | | 736.00 (472.00, 1,288.00) | | <0.001 |  |
| SII, median (IQR) | 438.80 (217.47, 788.14) | | 365.49 (208.76, 601.81) | | 355.59 (176.48, 558.93) | | 0.292 |  |
| NLR, median (IQR) | 2.17 (1.20, 3.34) | | 1.96 (1.01, 2.58) | | 1.90 (1.40, 2.47) | | 0.314 |  |
| PLR, median (IQR) | 118.49 (88.75, 187.50) | | 123.90 (80.92, 197.24) | | 123.41 (84.09, 176.00) | | 0.961 |  |
| CAR, median (IQR) | 0.97 (0.48, 2.12) | | 0.60 (0.22, 1.25) | | 0.95 (0.51, 2.15) | | 0.015 |  |
| PNI, median (IQR) | 45.45 (40.30, 51.25) | | 43.95 (40.05, 50.07) | | 42.42 (38.11, 47.00) | | 0.100 |  |
| CALLY, median (IQR) | 1.68 (0.84, 4.90) | | 2.82 (1.33, 9.60) | | 1.51 (0.65, 2.61) | | 0.010 |  |
| **Treatment regimen, n (%)** |  | |  | |  | | 0.244 |  |
| RIF + DOX | 17 (30.91) | | 27 (50.94) | | 17 (31.48) | |  |  |
| DOX or RIF + FQs | 3 (5.45) | | 3 (5.66) | | 5 (9.26) | |  |  |
| DOX + RIF + FQs | 28 (50.91) | | 21 (39.62) | | 28 (51.85) | |  |  |
| DOX + RIF + Ceph | 7 (12.73) | | 2 (3.77) | | 4 (7.41) | |  |  |
| Patients were categorized into tertiles according to pre-treatment IFN-γ levels: T1 ≤ 8.44 pg/mL, T2 > 8.44 to ≤ 28.22 pg/mL, and T3 > 28.22 pg/mL. Continuous variables are presented as mean ± SD or median (IQR), and categorical variables as n (%). P values were calculated using one-way ANOVA or the Kruskal–Wallis test for continuous variables and the χ² test or Fisher’s exact test for categorical variables, as appropriate. Abbreviations: WBC, white blood cell count; PLT, platelet count; Neu#, neutrophil count; Lym#, lymphocyte count; ALB, albumin; CRP, C-reactive protein; ESR, erythrocyte sedimentation rate; SF, serum ferritin; IFN-γ, interferon-gamma; SII, systemic immune-inflammation index; NLR, neutrophil-to-lymphocyte ratio; PLR, platelet-to-lymphocyte ratio; CAR, C-reactive protein-to-albumin ratio; PNI, prognostic nutritional index; CALLY, C-reactive protein–albumin–lymphocyte index. | | | | | | | |  |
